# Application of the modified Mini-CEX combined with the SBAR model in the experimental teaching of health assessment course: a quasi-experimental study

**DOI:** 10.1186/s12909-026-10086-0

**Published:** 2026-08-03

**Authors:** Le Wang, Miao Wang, Feng-ling Zhang, Ning Liu, Hui-xue Wang, Miao Yu, Shenglin Chen

**Affiliations:** 1 School of Nursing, Wannan Medical University, Wuhu, Anhui China; 2 Department of Hepatobiliary Surgery, Wuhu City Second People’s Hospital, No.6, Duchun Road, Wuhu, Anhui 241000 China

**Keywords:** Modified Mini-CEX, SBAR, Health assessment, Experimental teaching

## Abstract

**Objective:**

This study aimed to explore the effects of the modified Mini-Clinical Evaluation Exercise (Mini-CEX) combined with the Situation-Background-Assessment-Recommendation (SBAR) model in the experimental teaching of Health Assessment course.

**Background:**

The Mini-cex and the SBAR model are widely used in clinical nursing to improve nurses’ operational skills and general competence. However, there is a lack of evidence to indicate that their combination can produce a synergistic effect on undergraduates’ multiple abilities, such as autonomous learning and critical thinking.

**Design:**

Quasi-experimental.

**Methods:**

This study included 311 nursing students in two parallel classes at a nursing college and divided them into an intervention group (155 students) and a control group (156 students). In the intervention group, an modified Mini-CEX combined with the SBAR teaching method was adopted, and the teaching process included classroom scenario simulation and clinical apprenticeship. In the control group, conventional teaching methods were used. At the end of the course, the 2 groups of students were compared in terms of independent learning ability, critical thinking ability, nurse empathy, attitude toward communication skills, and course achievement.

**Results:**

After intervention, the total independent learning ability score(110.20±6.37 vs. 98.79±7.57), the total critical thinking ability score(295.43±15.34 vs. 272.52±13.85), the total empathy ability score(93.08±7.61 vs. 86.22±6.82), the positive scores of students' attitudes toward communication skills score(50.35±3.95 vs. 46.58±4.27), and the clinical skills assessment score(78.83±8.81 vs. 74.82±9.41) of the students in the intervention group were significantly higher than those of control group, and with a statistically significant difference (*p* <0.01). There was no statistically significant difference in the total theoretical examination score (*p* >0.05).

**Conclusion:**

The application of the modified Mini-CEX combined with the SBAR model in the experimental teaching of Health Assessment course had a positive effect on students, which can improve students' clinical practice abilities and comprehensive clinical quality. The model can be further promoted in the future to improve the quality of teaching and students' professional abilities.

## Background

Nursing is a comprehensive discipline with strong practical application. Although clinical teaching is integrated into the entire process of nursing specialty education, it should focus on nursing competence and combine theory and practice [1]. Health assessment is the process of systematically collecting and analyzing the health information of patients to identity their health status, existing health problems and possible causes, thereby determining nursing needs and formulating nursing diagnosis. Health assessment ability is one of the core abilities of nurses. Patients’ health problems can only be discovered and solved through health assessment [2]. The experimental teaching of Health Assessment course is a key link between the clinical work ability of nursing students.It serves as a bridge for nursing students to transition from theoretical learning to practical application, and plays a crucial role in cultivating nurses’ comprehensive abilities. The learning effect of Health Assessment will directly affect the students’ subsequent learning of clinical nursing courses as well as their ability to work in clinical nursing. Health Assessment plays a crucial role in fostering nursing students’ communication skills, critical thinking abilities, and clinical reasoning capacities. This not only facilitates students’ better adaptation to the requirements of nursing positions and enables them to perform clinical work more effectively but also helps them better meet the societal demand for high-quality nursing professionals. However, traditional experimental teaching and evaluation methods had certain limitations, such as the lack of effective assessment tools and an insufficient emphasis on communication skills, which cannot fully reflect the comprehensive quality and clinical practice ability of students.

The Mini-Clinical Evaluation Exercise (Mini-CEX) is an assessment tool for evaluating the clinical competence of residents and medical students that was developed and recommended by Norcini et al. of the American Academy of Internal Medicine. This implement tests the knowledge, skills, and attitudes of medical students in a real environment [3]. Yi developed the Nursing-Mini-CEX scale for nursing students via the Delphi method, which has good reliability and validity [4]. Applications in nursing clinical assessment and education have confirmed that the Mini-CEX can improve operational skills, comprehensive competence, and attention to humanistic care [5–7]. The Mini-CEX has the advantages of simplicity and an emphasis on overall efficiency. It focuses on feedback and has the function of teaching so that students can understand their strengths and weaknesses in a timely manner after the assessment to promote learning through examination. Considering the characteristics of students and courses, authors modified the Mini-CEX to better suit the teaching features of this study.

Communication is crucial for obtaining patient health information in Health Assessment. The Situation-Background-Assessment-Recommendation (SBAR) model is a standardized and structured communication model proposed by the World Health Organization (WHO). The current situation (Situation), the background and reasons for the situation (Background), the professional assessment of the situation (Assessment), and recommendations on how to solve the problem (Recommendation) are considered in the SBAR model [8]. The purpose of the SBAR method is to facilitate the effective transmission of patient information and ensure that healthcare professionals receive timely and accurate information, thereby enhancing the work efficiency of the team and safeguarding clinical safety [9]. Nurses’ ability to communicate clearly and concisely contributes to improving the quality of nursing care and minimizing potential accidents that may occur in clinical settings [10, 11]. The SBAR model is widely applied in clinical practice and healthcare education settings [12–14]. As a structured communication tool, it enables clear and efficient communication within a short period [15, 16]. In the context of Health Assessment, the SBAR model exhibits high applicability and practical value, as it facilitates more accurate and timely information transmission through standardized communication processes.

The Mini-CEX focuses on the comprehensive assessment of students’ clinical competencies, including history taking, physical examination, clinical judgment, professional attitude, and other core dimensions, and can provide targeted feedback on students’ weaknesses in clinical assessment. However, it lacks systematic guidance on structured communication, which may lead to students’ inability to effectively obtain and convey patient information during assessment. In contrast, the SBAR model emphasizes standardized, clear, and efficient communication, guiding students to convey patient information in a structured manner, which helps improve the accuracy and efficiency of information exchange.

In this study, the authors verified the feasibility of the modified Mini-CEX combined with the SBAR model in the experimental teaching of Health Assessment course, summarized the experience of its application and provided suggestions to improve the teaching method. It provides a basis for the reform of the experimental teaching of Health Assessment course and a reference for its application in other courses.

## Methods

### Study design and participants

Pilot testing phase: Conducted from February to June 2023, involving 140 nursing students from the School of Nursing at Wannan Medical University from the class of 2021(sophomores at that time), whose main purpose was to test the feasibility of the modified Mini-CEX scale and the intervention plan, adjust the teaching content and assessment criteria based on the pilot results, and optimize the intervention protocol.

Formal teaching intervention: Conducted from February to June 2024, a convenient cluster sampling method of second-year undergraduate nursing students of 2022 was recruited for this study. The sample inclusion criteria were as follows: (i) no previous experience in Health Assessment course; (ii) underwent basic medical courses as a freshman; (iii) had no prior experience in Mini-CEX and the SBAR model; (iv) provided voluntary informed consent to participate in the study.The exclusion criteria were: i) unable to fully participate in this study; ii)had learning disabilities that affect normal course.

The sample size was estimated based on the primary continuous outcome of this study. Based on the clinical skills assessment score of the pilot study subjects, the expected mean difference between the intervention and control groups in the primary outcome was estimated, and the corresponding standard deviations were used for sample size calculation. Using the two-sample t-test procedure in PASS software, with a two-sided significance level of 0.05 and a statistical power of 80% (1 − β = 0.80), the minimum required sample size was calculated to be 272 participants. Considering a potential attrition rate of 10%, the final required sample size was increased to 300. The total number of students in the two participating classes (*n* = 311) met the sample size requirement.

This study employed a quasi-experimental design. As the students in our study were from two parallel classes at one college, randomly assigning students to control their interactions with each other was impossible. Therefore, after comparing two parallel classes with no statistically significant differences in general information before the intervention, authors assigned the two parallel classes to the intervention and control groups. The control group received the conventional teaching method, and the intervention group employed an experimental teaching method based on the modified Mini-CEX combined with the SBAR model. Both groups of students took Health Assessment course in the second semester of their sophomore year.

### Interventions

The experimental teaching of Health Assessment was conducted in the second semester of the sophomore year, with a total of 68 credit hours, including 28 credit hours of classroom practical teaching and 40 credit hours of clinical apprenticeship teaching. Both groups of students used the fifth edition of Health Assessment published by People’s Medical Publishing House and the textbook Fundamentals of Nursing Laboratory published by the University of Science and Technology of China Press.

### The control group

The control group adopted the conventional teaching method. Each class was taught independently by a nursing instructor. Pre-class, the instructor provided the study tasks, materials and test questions in the online classroom for students to study on their own. In-class, the teacher introduced the case, demonstrated the operation and explained the key steps. Then, the students practiced. The teacher watched the students and provided guidance during the practice and summarized the practical training process before class ended. The training rooms were opened after class. In clinical probation teaching, the clinical nursing teachers created a probation plan according to the general probation teaching syllabus for undergraduate nursing students. The teaching plan included teaching-related theories and operational exercises in the department. The students learned about specific diseases and specialty nursing routines and strengthened their basic nursing practices.

### The intervention group

#### The modified Mini-CEX model

The intervention group was taught with the integrated modified Mini-CEX and the SBAR model. Since the study subjects were sophomores who had not begun their studies in internal medicine and surgery and did not have a deep understanding of the disease, the Nursing-Mini-CEX scale was not suitable for this study. Authors reviewed the relevant literature, combined the clinical competence assessment items and scoring criteria of the Nursing-Mini-CEX scale, and revised the teaching content which included inquiry, physical examination, nursing diagnosis (nursing problems), communication skills, humanistic care, organizational effectiveness and overall evaluation. Nursing inquiry was defined as guiding a patient in providing a comprehensive and focused account of their medical history without omitting important content. Physical examinations involved performing a focused physical examination for the patients’ condition and specific characteristics, requiring correct operation and a reasonable order. Nursing diagnosis (nursing problems) involved combining nursing assessment, physical examination, and test results to make appropriate nursing diagnoses for patients. The SBAR model was adopted for standardized communication. Humanistic care contained showing care and respect for the patient, with a kind and affectionate attitude, fully embodying humanistic care and taking care to protect the patient’s privacy. Organizational effectiveness was defined as a systematic and logical, well organized, concise and fluent process. Finally, authors evaluated whether each student’s process was standardized and proficient, appropriately measured, correctly performed, and completed on time.

#### Formation of the teaching team

Eight members with expertise in Health Assessment teaching were recruited as the core research and teaching team for this study. Specifically, the team consisted of 4 teachers from the School of Nursing (including 1 professor and 3 lecturers) and 4 clinical nursing experts from affiliated hospitals, who specialized in internal medicine, surgery, and emergency medicine respectively. All the participants were female and aged 33 to 51 (39.88 ± 5.96) years. The teaching experience ranged from 7 to 23 (13.50 ± 5.07) years. Among the members, 2 had senior (25%), 2 had associate senior (25%), and 4 had intermediate (50%) titles. All 8 faculty members were experienced in teaching and working and provided informed consent to participate in this study. The teaching team conducted a preliminary analysis of the learner and course characteristics, teaching environment, teaching resources and technical support through collective lesson preparation. Moreover, the necessary experimental equipment and teaching materials were prepared to ensure the safety and appropriateness of the teaching environment.

#### Development of the modified Mini-CEX model teaching nursing case base

Four case preparation groups were established. Each consisting of one full-time nursing faculty member and one clinical nursing expert. Each case was initially prepared by one faculty member and reviewed by another faculty member in the same group. Then cross-reviews were conducted among the teams, and finally submitted to the teaching team leader for review and finalization. All the cases were derived from real clinical cases and were used to test the students’ humanistic literacy by combining the relevant knowledge points of basic nursing and humanistic cultivation. The case bank covered common diseases in the emergency, internal medicine and surgery departments, such as drug-allergic shock, lobar pneumonia, chronic obstructive pulmonary disease, mitral stenosis in patient with rheumatic heart disease, acute myocardial infarction, cirrhosis of the liver, and acute gastrointestinal perforation.

#### The implementation of curriculum teaching

Pre-class, the teacher briefly explained the importance and purpose, basic concepts and implementation methods of the Mini-CEX and SBAR model and emphasized the precautions and requirements in laboratory teaching to the students. The teacher first conducted a demonstration to show the students how to use the modified Mini-CEX evaluation scale to assess the case and demonstrated the use of the SBAR model. The teacher divided the students into learning groups with 6 students each. The groups were established through social media platforms for teacher‒student communication, and posting clinical nursing cases, learning tasks, and operation videos to the group a week before class for students to prepare through team-based learning. In-class, under the joint guidance of nursing instructors and clinical nursing specialists, students completed a modified Mini-CEX combined with the SBAR model through clinical case studies and teamwork. This included disease observation, medical history collection, physical examination, role-playing, and the exchange of information and reports using the SBAR communication model. Finally, the two faculty members conducted assessments, provided feedback on the performance of the learning group in the classroom and made suggestions for improvement. The students were encouraged to ask questions and make comments. The teachers modified and refined the teaching methods and content on the basis of the students’ performance and feedback. The students reflected on their performance and summarized their learning to improve their clinical practice ability and comprehensive quality. Post-class, the teacher provided the test questions and opened an operation training room to strengthen the students’ operational skills.

Clinical probation was conducted based on the modified Mini-CEX and the SBAR model. Combined with specific clinical cases, probational students were arranged to participate in real clinical work, including patient reception, condition assessment, and nursing diagnosis. They were required to proactively apply the SBAR model in practice to communicate effectively with mentors and other members of the medical team. Clinical instructors provided timely feedback on the probational students’ performance and put forward specific improvement suggestions for their deficiencies in practical application. After the completion of probation, the students were organized to share and summarize their experiences.

#### Standardized Patient(SP)

Since the teaching required a strong clinical situation, this study uses a Standardized Patient(SP) [17]. To meet the clinical teaching needs of nursing students, our college arranged for four backbone teachers to participate in SP teacher training at institutions with relevant training qualifications. All four teachers had obtained SP trainer certification and were qualified to conduct SP recruitment-level training in accordance with relevant requirements. SPs selected included 30 undergraduate nursing students who were taking Health Assessment course from the class of 2020 in our school. After systematic training according to SP training requirements, 30 nursing students could accurately demonstrate the corresponding clinical symptoms and some signs of patients with related diseases. Thirty simulated patients, 17 males (57%) and 13 females (43%), aged 19–21 (19.93 ± 0.69) years, were informed and agreed to participate in this study.

#### Ethical considerations

This study was approved by the Ethics Committee of Wannan Medical College (No. 20240001). Each student who participated in this study provided informed consent and reserved the right to withdraw from the study at any time. To protect participants’ confidentiality, information about their identities was not collected, and all responses were anonymous.

### Instruments

#### General information questionnaire

General information included students’ age, gender, geographic region of origin, and academic performance in the previous semester.

#### Mini-Clinical Evaluation Exercise (Mini-CEX)

Yi used the Delphi method to develop the Nursing-Mini-CEX scale for nursing students. The Cronbach’s alpha coefficient of the scale was 0.780, and the split-half reliability coefficient was 0.842 [4]. Considering the characteristics of Health Assessment course, the Nursing-Mini-CEX was modified for three parts. Part one includes basic information, such as the time and place of the assessment, the diagnosis of the patient selected for the assessment, the patient’s condition, the results of the initial or follow-up examination, and the focus of the assessment. Part two includes seven scoring items, such as the nursing inquiry, physical examination, nursing diagnosis, communication skills, humanistic care, organizational effectiveness, and overall evaluation items. Each item is scored via a 9-point scale, with 1 to 3 points indicating noncompliance, 4 to 6 points indicating that the requirements are met, and 7 to 9 points indicating excellence. Part three contains feedback information, including the time used for observation and feedback, the teacher’s satisfaction with the assessment, and the teacher’s specific suggestions for the student.

#### Self-directed learning ability

The self-directed learning ability assessment scale developed by Zhang [18] was used to evaluate the self-directed learning ability. This scale includes 4 primary indicators with a total of 30 items: learning motivation (8 items), self-management ability (11 items), cooperation ability (5 items), and information literacy (6 items). A 5-point Likert rating system was adopted, with higher scores indicating stronger self-directed learning ability. The Cronbach’s alpha coefficient of this scale was 0.822, and the total score half coefficient was 0.788.

#### Critical thinking ability

The Chinese Critical Thinking Disposition Inventory (CTDI-CV) revised by Peng [19] was used to measure the critical thinking ability. The scale includes 70 items and 7 aspects: searching for truth, open-mindedness, analytical ability, systematicity, self-confidence in critical thinking, curiosity, and cognitive maturity. Responses were scored on a 6-point Likert-type scale ranging from 1 = “strongly disagree” to 6 = “strongly agree”. The content validity coefficient of the scale was 0.90, and the validity coefficients of each subcategory ranged from 0.54 to 0.77.

#### Nursing student empathy

The Jefferson Scale of Empathy for Nursing Students (JSPE-NS) [20] translated by Qiu was used to investigate the empathic ability of the nursing students. The JSPE-NS comprises 20 items and three subscales, including viewpoint selection, emotional care, and transpersonal thinking. Each item is scored on a 7-point Likert scale ranging from 1 = “completely disagree” to 7 = “completely agree”. The higher the score is, the higher the level of empathy. The Cronbach’s α coefficient of the scale was 0.739, and the retest reliability coefficient was 0.843.

#### Attitude toward communication skills

The Chinese version of the Communication Skills Attitude Scale (Ch-CSAS) [21] was used to investigate the nursing students’ attitudes toward communication skills. The Ch-CSAS consists of 13 questions each on the Positive Attitude Scale (PAS) and the Negative Attitude Scale (NAS). Each item is rated on a 5-point Likert scale ranging from strongly disagree to strongly agree, with scores ranging from 1 to 5. The higher the PAS the better, and the lower the NAS the better. The total Cronbach’s alpha coefficient of the scale was 0.841, and the retest reliability coefficient was 0.894.

#### Course achievement

The theoretical assessment content was taken from the textbook, and the test was independently developed by our teaching and research department. The assessment of operational skills involved a physical examination conducted by the students, with a total score of 100 points.

#### Data collection

After the course ends, a questionnaire was administered to the study participants in the intervention and control groups via “Wenjuanxing” (https://www.wjx.cn/), with detailed instructions on the purpose and method of completing the questionnaire. The students were informed that the survey was anonymous and that the results of the study would only be used to objectively assess teaching and learning effectiveness. The quality of the study was ensured by setting the time for online completion, the number of submissions and the criteria for submission.

#### Data analysis

The data were analyzed via SPSS software (version 23). Continuous variables were described by mean and standard deviation, and categorical variables were described by frequency and percentage. The chi-square test and t-test were used to compare the two groups. A difference considered statistically significant at *p* < 0.05 was taken into consideration.

## Results

### Characteristics of students

There were 156 students in the control group, including 35 males and 121 females; their ages ranged from 19 to 21 years (19.92 ± 0.77 years). In the intervention group, there were 155 students, including 36 males and 119 females; their ages ranged from 19 to 21 years (19.83 ± 0.80 years). There was no statistically significant difference in the baseline data between the two groups of students (*p* > 0.05).In the exams at the end of last semester, there was no statistically significant difference in the theoretical and practical scores between the two groups of students (*p* > 0.05).

### Comparison of the self-directed learning ability of the two groups of students

The results revealed that the total score and the scores of the dimension of self-directed learning ability of the students in the intervention group were significantly higher than those of the students in the control group and with a statistically significant difference (*p* < 0.01) (Table 1).

**Table 1:** Comparison of self-directed learning ability

Groups	Learning motivation	Self-management ability	Cooperation ability	Information literacy	Total
Intervention group (*n* = 155)	29.40 ± 3.08	40.31 ± 3.88	18.17 ± 2.52	22.29 ± 2.53	110.20 ± 6.37
Control group (*n* = 156)	26.22 ± 3.67	35.95 ± 4.46	16.22 ± 2.71	20.40 ± 3.27	98.79 ± 7.57
*t*	8.256	9.196	6.596	5.711	14.378
*p*	0.000	0.000	0.000	0.000	0.000

### Comparison of critical thinking skills between the two groups

Following the intervention, there were statistically significant differences in the total score and the scores of the dimension of critical thinking skills (*p* < 0.01) between the intervention and control groups (Table 2).

**Table 2:** Comparison of the critical thinking skills of the two groups

Groups	Searching for truth	Open-mindedness	Analytical ability	Systematicity	Self-confidence in critical thinking	Curiosity	Cognitive maturity	Total
Intervention group (*n* = 155)	40.31 ± 4.32	42.93 ± 3.44	41.86 ± 4.05	41.26 ± 5.47	41.63 ± 4.37	43.19 ± 3.70	43.42 ± 4.41	295.43 ± 15.34
Control group (*n* = 156)	38.08 ± 3.29	39.37 ± 2.91	38.16 ± 3.73	37.27 ± 2.50	38.76 ± 4.47	40.56 ± 5.62	40.25 ± 4.67	272.52 ± 13.85
*t*	5.114	9.858	8.376	8.036	5.722	4.873	6.152	13.819
*p*	0.004	0.000	0.001	0.002	0.000	0.000	0.000	0.000

### Comparison of empathy skills between the two groups

The intervention group showed significantly better viewpoint selection(*t* = 6.134, *p* < 0.01), emotional care(*t* = 3.996, *p* < 0.01) and transpersonal thinking(*t* = 8.187, *p* < 0.01) compared to the control group(Table 3).

**Table 3:** Comparison of empathy skills between the two groups

Groups	Viewpoint selection	Emotional care	Transpersonal thinking	Total
Intervention group (*n* = 155)	45.66 ± 4.50	36.71 ± 4.72	10.71 ± 1.70	93.08 ± 7.61
Control group (*n* = 156)	42.40 ± 4.84	34.73 ± 3.98	9.09 ± 1.79	86.22 ± 6.82
*t*	6.134	3.996	8.187	8.365
*p*	0.000	0.000	0.000	0.000

### Comparison of attitudes toward communication skills between the two groups

Post-intervention, the positive score of students’ attitudes toward communication skills in the intervention group was significantly higher than those in the control group (50.35 ± 3.95 vs. 46.58 ± 4.27, *t* = 8.086, *p* < 0.01), the negative score was lower than those in the control group(32.32 ± 4.155 vs. 35.56 ± 5.47, *t*=-5.903, *p* < 0.01) (Table 4).

**Table 4:** Comparison of attitudes toward communication skills between the two groups

Groups	PAS	NAS
Intervention group (*n* = 155)	50.35 ± 3.95	32.32 ± 4.15
Control group (*n* = 156)	46.58 ± 4.27	35.56 ± 5.47
*t*	8.086	-5.903
*p*	0.000	0.000

### Comparison of the course achievement of the two groups

In terms of course achievement, the intervention group’s clinical skills assessment score (78.83 ± 8.81 vs. 74.82 ± 9.41, *t* = 4.541, *p* < 0.01) was higher than the control group, but there was no statistically significant difference in theoretical exam score(72.68 ± 8.67 vs. 73.21 ± 8.82, *t* = 5.532, *p* = 0.595) (Table 5).

**Table 5:** Comparison of the assessment scores of the two groups

Groups	Theoretical examination	Clinical skills assessment
Intervention group (*n* = 155)	72.68 ± 8.67	78.83 ± 8.81
Control group (*n* = 156)	73.21 ± 8.82	74.82 ± 9.41
*t*	5.532	4.541
*p*	0.595	0.000

## Discussion

This study revealed that the total independent learning ability score and the scores of each dimension, the total critical thinking ability score and the scores of each dimension, the total empathy ability score and the scores of each dimension, the attitudes toward communication skills score, and the clinical skills assessment score of the students in the intervention group were significantly higher than those in the control group, and with a statistically significant difference (*p* < 0.01). The application of modified Mini-CEX combined with the SBAR model was associated with favourable learning outcomes in the experimental teaching of Health Assessment, as well as higher self-reported clinical practice readiness and overall comprehensive literacy among nursing students. This may be because the modified Mini-CEX combined with the SBAR model allows for a more comprehensive evaluation of students’ abilities, including clinical observation, history taking, physical examination, attitudes toward communication skills, and humanistic care. In addition, the SBAR model could help students better adapt to clinical work, improve their attitudes toward communication skills, and strengthen their abilities in teamwork and standardized procedures. These abilities are highly important for students’ future career development. To meet the demand for nursing professionals and advance the development of healthcare, the training system for highly educated nurses requires continuous optimization, and a scientific training model should be established. As a backup force for nursing professionals, the core competencies of undergraduate nursing students are directly related to the stability of the nursing team and the level of clinical nursing quality. As a result, developing nursing students’ core competences has emerged as a critical teaching aim and research priority for nursing educators [22]. The Mini-CEX assessment tool focuses on evaluating the clinical thinking ability of medical students [23, 24], whereas the SBAR communication model strengthens the ability of students to think logically and express their thoughts clearly. Therefore, the combination of the two could enable students to be more organized and focused when evaluating patients.

This study investigated participants’ critical thinking ability. The results demonstrated that nursing students’ the total critical thinking ability score and the scores of each dimension were significantly higher than those in the control group. Motefakker et al. [25] reported that applying the Mini-CEX to nursing teaching positively affected students’ clinical competence and professionalism. This study demonstrated that integration of the SBAR model enabled students to better comprehend patients’ conditions and organize and convey relevant information more systematically, thereby enhancing the accuracy and comprehensiveness of their clinical assessments. Furthermore, the Mini-CEX was combined with the SBAR model through role-play and case presentations. This approach was consistent with clinical nursing practice and allowed nursing students to refine their professional knowledge, technical skills, communication, teamwork, and humanistic care competencies [25]. In this study, the Mini-CEX assessment tool and SBAR model were used by the instructors to understand the students’ learning in real time, identify problems and provide targeted guidance. Moreover, by evaluating students’ performance and assessment outcomes, teachers could identify deficiencies in clinical reasoning and communication skills and provide timely feedback and targeted guidance. Consistent with the findings of this study, teaching methods and content can be continuously adjusted and optimized based on students’ performance and feedback, so as to further enhance teaching quality [26]. Collaborative teaching between nursing faculty and clinical nurse specialists on the basis of clinical nursing cases could help students integrate theoretical knowledge with clinical nursing practice and develop logical thinking.

Nursing students should develop practical nursing abilities and communication skills through theoretical learning and clinical practice [27]. At present, many nursing students were passive observers in practical classroom teaching and clinical internships and lacked active practical operation. Moreover, nursing students were unable to clearly convey information because they could not apply theoretical knowledge to nursing practice and lacked experience in communicating with other healthcare professionals [8]. The SBAR communication model is a standard communication method that emphasizes the accuracy and organization of information delivery and is used to convey information accurately and effectively [12, 28]. In the experimental teaching of Health Assessment course, the use of the SBAR communication model in this study helped students better communicate with patients and obtain more accurate information. Accordingly, both assessment accuracy and students’ attitudes toward communication skills were enhanced. Classroom simulation teaching served as a valuable supplement to clinical probation. It allows students to role-play nurses, patients, family members, and other stakeholders, and to repeatedly practice using standardized communication tools in simulated scenarios. With the modified Mini-CEX combined with the SBAR model, students demonstrated improved teamwork, clearer role definition and task allocation, and more effective communication and information sharing, thereby enhancing overall team efficiency and effectiveness. The results of the study revealed that the Mini-CEX combined with the SBAR model enabled nurses to identify changes in patients’ conditions more accurately, thereby facilitating precise, effective communication and collaboration among healthcare professionals [29]. Adaptation to clinical practice is critical for novice nurses entering clinical settings after graduation [30]. SBAR-based education can improve students’ critical thinking when they justify their clinical judgments, identify optimal decisions, and verify them with evidence. This was consistent with the findings of Lancaster [31].

This study showed that the modified Mini-CEX combined with the SBAR model was conducive to improving the quality of teaching and the academic performance of nursing students in Health Assessment course. Classroom scenario simulation requires students to master solid theoretical knowledge and proficient clinical skills, thereby placing higher demands on their teamwork, clinical reasoning, and communication abilities [32, 33]. Through teaching modes such as scenario simulation, group learning, and role-playing, students’ independent learning, critical thinking, empathy, and clinical reasoning abilities were improved.Students were able to flexibly apply theoretical knowledge and clinical reasoning to solve clinical nursing problems in both theoretical examinations and practical skill assessments.Case-based learning helps students develop comprehensive competencies and transfer knowledge to clinical nursing practice to identify and resolve complex health problems [34]. The integration of authentic workplace insights into classroom scenario-based simulation teaching, together with repeated practice in a low-stress environment, enhanced students’ confidence in clinical decision-making and improved their health assessment competence. Kim [32] concluded that simulation-based nursing educational interventions exerted a strong educational effect on students, particularly in the psychomotor domain. This finding was consistent with those reported by Lee [35] and Liu et al. [36], who investigated the effects of team-based learning and case-based teaching on undergraduate nursing students’ competencies.

## Conclusion

This study demonstrated that the modified Mini-CEX combined with the SBAR model presented promising application value in the experimental teaching of the Health Assessment course. Furthermore, this teaching approach was associated with improved students’ self-reported independent learning capacity, critical thinking, empathy, clinical reasoning, and communication attitudes, as well as enhanced teamwork and interdisciplinary communication awareness. Collectively, these findings provide novel insights and a practical reference for the reform and innovation of nursing education.

### Limitations

This study has several limitations that should be acknowledged. First, all participants were recruited from a single grade of one educational institution using convenient cluster sampling. This single-center sampling approach restricts sample representativeness and may limit the external generalizability of the current findings. Second, this investigation only included a relatively narrow range of assessment indicators, which failed to fully capture nursing undergraduates’ comprehensive literacy and overall clinical competence. Future studies are recommended to adopt multi-dimensional evaluation systems to comprehensively evaluate students’ learning outcomes and clinical practical capabilities. Third, the revised Mini-CEX scale developed for this integrated teaching programremains unpublished and lacks complete, formally validated psychometric properties, including systematic reliability and validity testing. This constitutes a notable methodological limitation of the present study. Subsequent research will expand the sample size to complete rigorous scale validation and report full psychometric data in a specialized methodological study. Fourth, this study adopted an integrated teaching intervention that combines the modified Mini-CEX and SBAR model. The current study design could not disentangle the independent instructional effect of each component, making it impossible to quantify the unique contribution of Mini-CEX versus SBAR to the observed improvements in student performance. Separated controlled trials focusing on individual teaching modules are warranted in future research to clarify their respective values. Fifth, the combined teaching framework imposes high requirements on instructors, who need proficient case-writing skills, professional classroom management ability, and solid clinical nursing experience. Heterogeneity in teachers’ comprehensive competency may lead to inconsistent teaching delivery and interfere with the stability of student learning outcomes. Standardized faculty training and unified assessment protocols are essential to ensure consistent and stable implementation of this teaching model. Sixth, although both Mini-CEX and SBAR emphasize structured feedback, this study did not establish a systematic and sustainable feedback improvement mechanism throughout the teaching process. Given the above limitations, educators should fully recognize the applicable boundaries and inherent shortcomings of this combined teaching mode. Teaching strategies and evaluation schemes can be flexibly adjusted according to teaching objectives, student characteristics, and available educational resources. Further optimization of feedback systems, teacher training procedures, and multi-faceted evaluation frameworks can improve the effectiveness and long-term sustainability of the intervention, thereby providing credible evidence for clinical nursing curriculum reform and supporting the cultivation of high-quality nursing talents.

## Data Availability

The datasets used and/or analyzed during the current study are available from the corresponding author upon reasonable request.
